# Oncostatin M (OSM) protects against cardiac ischaemia/reperfusion injury in diabetic mice by regulating apoptosis, mitochondrial biogenesis and insulin sensitivity

**DOI:** 10.1111/jcmm.12501

**Published:** 2015-03-08

**Authors:** Dongdong Sun, Shuang Li, Hao Wu, Mingming Zhang, Xiaotian Zhang, Liping Wei, Xing Qin, Erhe Gao

**Affiliations:** a Department of Cardiology, Xijing Hospital, Fourth Military Medical UniversityXi'an, China; b Department of Toxicology, School of Public Health, Fourth Military Medical UniversityXi'an, China; c Department of Cardiology, Tianjin Union Medicine CenterTianjin, China; d Center for Translational Medicine, Temple University School of MedicinePhiladelphia, PA, USA

**Keywords:** oncostatin M, ischaemia/Reperfusion injury, inositol pyrophosphate 7, diabetes

## Abstract

Oncostatin M (OSM) exhibits many unique biological activities by activating Oβ receptor. However, its role in myocardial I/R injury in diabetic mice remains unknown. The involvement of OSM was assessed in diabetic mice which underwent myocardial I/R injury by OSM treatment or genetic deficiency of OSM receptor Oβ. Its mechanism on cardiomyocyte apoptosis, mitochondrial biogenesis and insulin sensitivity were further studied. OSM alleviated cardiac I/R injury by inhibiting cardiomyocyte apoptosis through inhibition of inositol pyrophosphate 7 (IP7) production, thus activating PI3K/Akt/BAD pathway, decreasing Bax expression while up-regulating Bcl-2 expression and decreasing the ratio of Bax to Bcl-2 in db/db mice. OSM enhanced mitochondrial biogenesis and mitochondrial function in db/db mice subjected to cardiac I/R injury. On the contrary, OSM receptor Oβ knockout exacerbated cardiac I/R injury, increased IP7 production, enhanced cardiomyocyte apoptosis, impaired mitochondrial biogenesis, glucose homoeostasis and insulin sensitivity in cardiac I/R injured diabetic mice. Inhibition of IP7 production by TNP (IP6K inhibitor) exerted similar effects of OSM. The mechanism of OSM on cardiac I/R injury in diabetic mice is partly associated with IP7/Akt and adenine mononucleotide protein kinase/PGC-1α pathway. OSM protects against cardiac I/R Injury by regulating apoptosis, insulin sensitivity and mitochondrial biogenesis in diabetic mice through inhibition of IP7 production.

## Introduction

Cardiovascular complications are responsible for the leading cause of death among patients with diabetes 1. Diabetes is now considered to be a risk equivalent of coronary artery disease for future MI and cardiovascular death 2. The presence of diabetes has a negative impact on the outcome of the patients with acute coronary syndromes 3. As a result of the exposure to abnormal substrate and cytokines, our previous studies have demonstrated that the myocardium of diabetic patients is more vulnerable to cardiac I/R injury than those individuals without diabetes 4,5. However, the effective strategies which can reduce cardiac I/R injury in diabetic conditions are not well developed.

Mitochondria play a key role in diabetes and cardiac I/R injury by regulating energy homoeostasis and is emerging as a key target in cardiometabolic disease therapy 6. Cardiac function maintenance is critically dependent on mitochondrial oxidative phosphorylation as a major source of adenosine triphosphate (ATP) 7. Cardiac mitochondria represent a key actor of the biological systems addressed to prevent any mismatch between ATP production and utilization, and thus any major disruption in the energy available for cardiac function 8. Abundant evidence have suggested that mitochondrial dysfunction is also a main cause of insulin resistance, one of the main characteristics of type 2 diabetes, and diabetes related cardiac comorbidities 9. Factors which contribute to mitochondrial dysfunction, such as mitochondrial biogenesis and oxidative stress, can also lead to insulin resistance in different insulin-target tissues, and its association with mitochondrial dysfunction can culminate in the development of cardiovascular diseases 6. A growing number of studies confirm that modulating mitochondrial survival/cell-death pathways will subsequently affect cardiomyocyte apoptosis and necrosis 7. In this regard, therapies that enhance mitochondrial biogenesis may increase insulin sensitivity as well as decrease cardiomyocyte apoptosis and necrosis during I/R injury in diabetic mice.

Oncostatin M (OSM), produced by activated T lymphocytes, monocytes and macrophages, is an inflammatory cytokine that belongs to the interleukin-6 (IL-6) class of cytokines 10. OSM exerts a variety of physiological and pathophysiological functions such as inflammation, tissue remodelling and cell growth 11. OSM induces stromal cell derived factor 1 and VEGF in cardiomyocytes, modulates extracellular matrix degradation and is also involved in the modulation of smooth muscle cell proliferation 12–14. Kubin *et al*. 15 demonstrated that inhibition of OSM signalling suppressed cardiomyocyte remodelling, resulting in deterioration of heart function after MI. Inositol pyrophosphate 7 (IP7), formed by a family of IP6Ks, represents a physiological inhibitor of Akt which mediates survival signal 16. Our previous study indicated that Akt signalling was impaired in diabetic mice underwent cardiac I/R injury 17. The effects of Akt inhibition by IP7 was also approved in our previous study 18. However, no efforts have been made to investigate (*i*) whether OSM protects against cardiac I/R injury in diabetic mice and (*ii*) the underlying mechanisms responsible for OSM in modulating myocardial I/R injury in diabetic mice.

## Research design and methods

### Animals

The experiments were performed in adherence with the National Institutes of Health Guidelines on the Use of Laboratory Animals and were approved by the Fourth Military Medical University Ethic Committee on Animal Care (Approval ID: 2010022). Male db/db were obtained from Jackson Laboratories (Bar Harbor, ME, USA). We used db/db mice at the age of 12–16 weeks (50–60 g) when they had developed overt diabetes.

Db/db mice were randomly allocated into the following groups with *n* = 30 each: (*i*) Db/db + sham group (Sham); (*ii*) Db/db + I/R group (I/R); (*iii*) Db/db + OSM + I/R group (OSM); (*iv*) Db/db + TNP + I/R (TNP) and (*v*) Db/db + TNP + OSM + I/R (TNP + OSM). Before constructing cardiac I/R model, OSM (60 ng/g; ≥97%, Sigma Aldrich, St. louis, MO, USA) was injected intraperitoneally for 14 days. TNP (IP6K inhibitor, 10 mg/kg; ≥95%, Sigma-Aldrich) was injected *via* the tail vein for 14 days. TNP + OSM group received TNP injection 10 min. before OSM injection for 14 days. All the reagents were dissolved in DMSO. The sham group and the I/R group received the same volume of DMSO for 14 days. The time interval between the last injection and ischaemia-reperfusion was 1 hr. Blood glucose concentration was determined by using a reflectance metre (Accu-Chek, Roche Diagnostics GmbH, Mannheim, Germany). Food intake and bw were recorded regularly.

129-Osmr^tm1.1Nat^/J mice were purchased from Jackson Laboratories which possess loxP sites on either side of the second exon (first coding exon) in the OSM receptor (Oβ) gene. 129-Osmr^tm1.1Nat^/J mice were crossed with C-Tg(CMV-cre)1Cgn/J mice (Jackson Laboratories) to knockout OSM receptor Oβ. Real-time PCR (RT-PCR) was used to screen Oβ^−/−^ mice and Oβ^+/+^ mice.

Diabetes was induced in male Oβ^−/−^ and Oβ^+/+^ mice by intraperitoneal injections (i.p.) of STZ (50 mg/kg, STZ was dissolved in 0.1 M citrate buffer, pH 4.5) as previously described 19. All the mice were fed with high glucose and high-fat diet after STZ injection. Blood glucose concentration was determined every week after STZ injection. Random blood glucose was tested in each mouse for three times. All of these three values ≥16.7 mmol/l were considered as a cut-off point for diabetes. Two months after STZ administration, diabetic Oβ^−/−^ mice and diabetic Oβ^+/+^ mice were subjected to myocardial I/R injury.

### Construction of I/R injury animal model and hemodynamic evaluation

I/R injury animal model was constructed by LAD ligation for 30 min. followed by 3 hrs reperfusion as previously described 20. Cardiac function was determined by invasive hemodynamic evaluation methods as previously described 20.

### Measurement of myocardial infarct size

Myocardial infarct size was evaluated by Evans Blue/TTC staining as previously described 20.

### Determination of myocardial apoptosis

Myocardial apoptosis was determined by terminal deoxynucleotidyl transferase-mediated dUTP-biotin nick end labelling (TUNEL) staining and caspase-3 activity assay as previously described 20.

### Determination of cardiac function

Echocardiography was conducted at 24 hrs after I/R injury as previously described 20.

### Mitochondrial calcium retention capacity

The mitochondrial calcium retention capacity (mCRC) was determined as the capacity of mitochondria to uptake calcium before permeability transition, to test the sensitivity of the mitochondrial permeability transition pore (mPTP) opening to calcium.

### ROS production and Manganese superoxide dismutase activity

The production of Reactive oxygen species (ROS) was measured in frozen tissue by electron paramagnetic resonance (EPR) spectroscopy according to Mellin's methods 21. The ROS levels were expressed in arbitrary units per milligram of wet tissue. Manganese superoxide dismutase (MnSOD) was assayed as Vives-Bauza has previously described 22 and expressed in unit/mg.

### *In vitro* citrate synthase, chain complex activities and ATP content

Citrate synthase (CS) and electron transport chain complex activities (Complex I, II, III, IV and IV) were measured using a commercially available CS activity assay kit (Sigma-Aldrich). The ATP content of the myocardium was measured using an ATP bioluminescent assay kit (Sigma-Aldrich) according to the standard protocols.

### Statistical analysis

Continuous variables that approximated the normal distribution were expressed as means ± SD. Comparison between groups were subjected to anova followed by Bonferroni correction for *post hoc t*-test. Data expressed as proportions were assessed with a chi-squared test. Two sided tests have been used throughout, and *P* < 0.05 were considered statistically significant. SPSS software package version 14.0 (SPSS, Chicago, IL, USA) was used for data analysis.

## Results

### OSM administration alleviates cardiac I/R injury in db/db mice by inhibition of IP7 production

**Figure 1:**
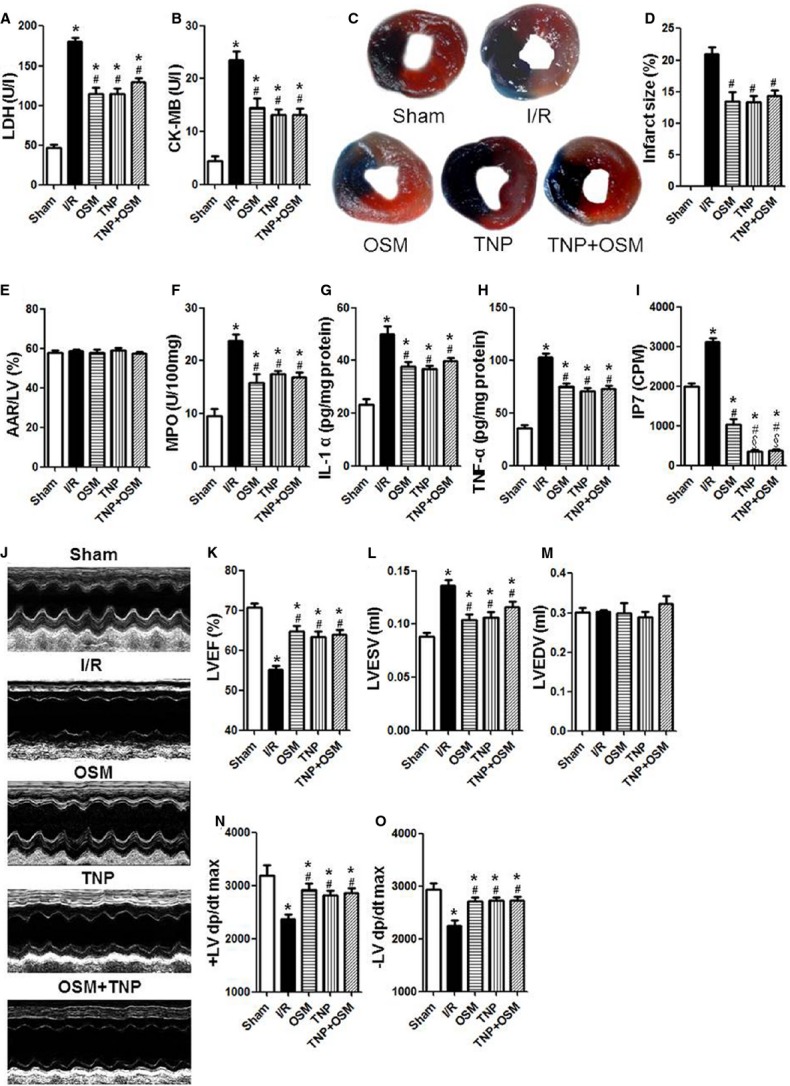
OSM protected against cardiac I/R injury in db/db mice by inhibition of IP7 production. (A and B) LDH and CK-MB release after myocardial I/R injury in db/db mice. (C) Representative images of infarct size as stained by Evans Blue and TTC. (D and E) Quantitative analysis of infarct size and AAR/LV at 3 hrs after I/R injury in db/db mice. (F) MPO activity at 3 hrs after I/R injury. (G and H) IL-1α and TNF-α release measured by enzyme-linked immunosorbent assay (ELISA). (I) IP7 levels evaluated by HPLC. (J) Representative images of echocardiography at 24 hrs after I/R injury. (K–M) LVEF, LVESV, LVEDV measured by echocardiography. (N and O) The ±LV dp/dt max obtained by hemodynamic evaluation 3 hrs after I/R injury. LDH, Lactate dehydrogenase; CK-MB, creatine kinase-MB; AAR, area at risk; LV, left ventricle; MPO, myeloperoxidase; IL-1α, interleukin-1α; TNF-α, tumour necrosis factor-α; IP7, inositol pyrophosphate 7; HPLC, High performance liquid chromatography; LVEF, Left ventricular ejection fraction; LVESV, Left ventricular end-systolic volume; LVEDV, Left ventricular end-diastolic volume. The columns and errors bars represent means and SD. *P < 0.01 *versus* Sham, ^#^*P* < 0.01 *versus* I/R, ^§^*P* < 0.01 *versus* OSM.

Lactate dehydrogenase (LDH) and creatine kinase-myocardium isoenzyme (CK-MB), biochemical markers of myocyte injury, were significantly decreased in the OSM treated group as compared with the I/R group (Fig.1A and B). Representative images of infarct size as stained by Evans Blue and TTC were shown in Figure1C. OSM administration significantly decreased infarct size at 3 hrs after I/R injury compared with the I/R group (Fig.1D). The ratio of area at risk (AAR) to left ventricle (LV) area had no statistical difference between groups, indicating that LAD ligature was reproducibly performed at the same level (Fig.1E). OSM treatment resulted in a noticeable decrease in MPO activity, IL-1α and TNF-α release and IP7 production compared with the I/R group (Fig.1F–I). Echocardiography evaluated at 24 hrs after I/R injury revealed that OSM significantly enhanced LVEF, decreased LVESV as compared with the I/R group (Fig.1J–M). Hemodynamic measurements performed at 3 hrs after I/R injury indicated that the ±LV dp/dt max were increased in the OSM group as compared with the I/R group (Fig.1N and O).

TNP, inhibitor of IP6Ks, blocks the production of IP7. TNP pretreatment exhibited similar results of OSM administration: decreased LDH and CK-MB release (Fig.1A and B), decreased infarct size (Fig.1C–E) and MPO activity (Fig.1F), reduced IL-1α and TNF-α release (Fig.1G and H), and blocked IP7 production (Fig.1I). Furthermore, TNP administration also improved cardiac function as evaluated by LVEF (Fig.1J and K) and ±LV dp/dt max (Fig.1N and O).

To determine the causative role of OSM/IP7 signalling in cardiac I/R injury, we treated the db/db mice systemically with TNP followed by OSM before cardiac I/R injury. Interestingly, OSM did not exhibit additional protective effects against cardiac I/R injury as compared with the TNP treated group.

### Oβ (OSM receptor) knockout exacerbates myocardial I/R injury, increases IP7 production in diabetic mice

**Figure 2:**
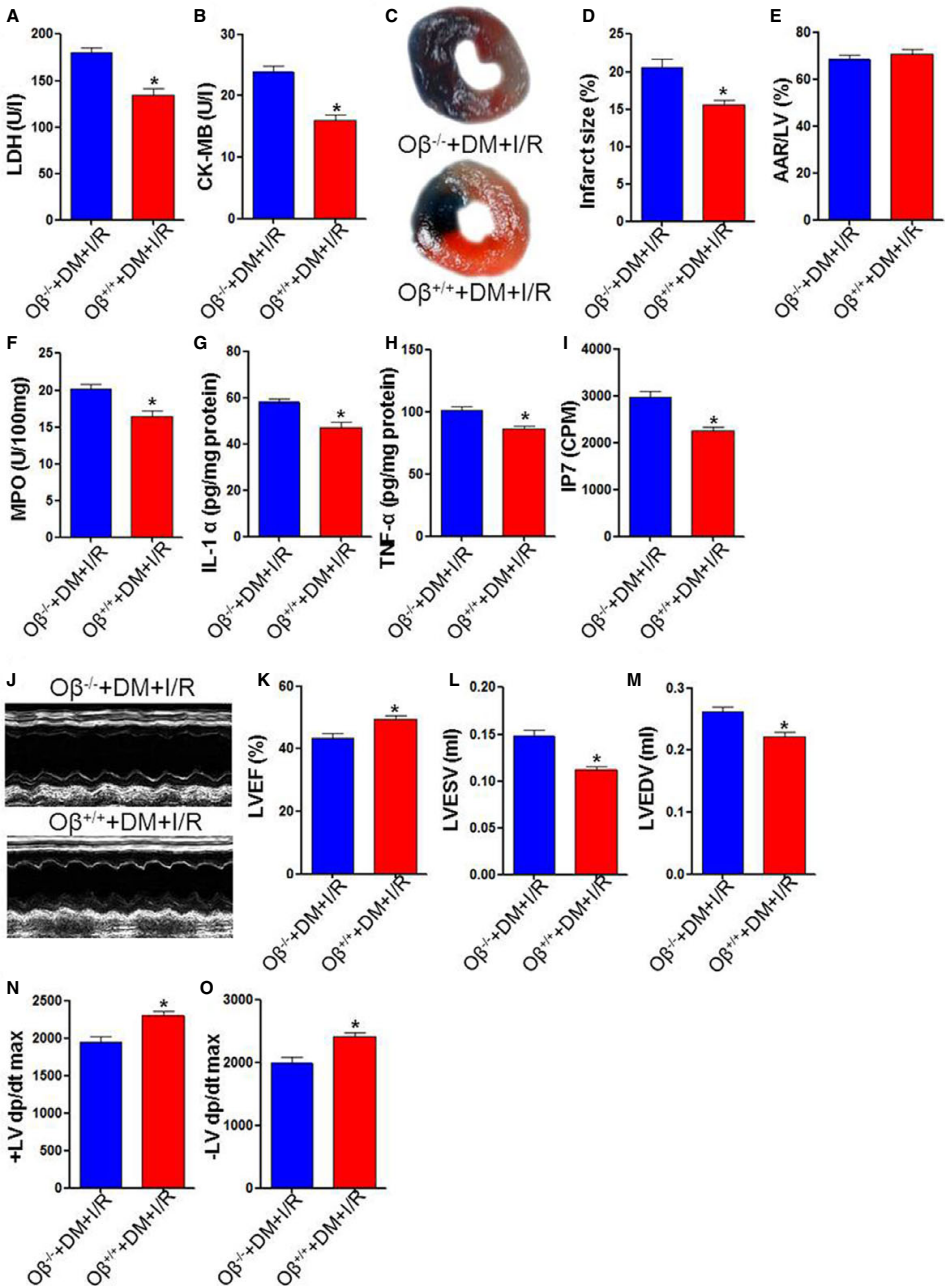
Oβ (OSM receptor) knockout exacerbated myocardial I/R injury. (A and B) Diabetic Oβ^−/−^ mice exhibited increased LDH and CK-MB release. (C) Representative images of infarct size as stained by Evans Blue and TTC. (D and E) Infarct size was smaller in the Oβ^+/+^ group. AAR/LV had no statistical difference between groups 3 hrs after I/R injury. (F–H) Diabetic Oβ^−/−^ mice had increased MPO activity, increased IL-1α and TNF-α release 3 hrs after cardiac I/R injury. (I) IP7 levels was higher in the Oβ^−/−^ group. (K) Representative images of echocardiography at 24 hrs after cardiac I/R injury. (J–L) Oβ knockout significantly decreased LVEF, increased LVESV and LVEDV. (M and N) ±LV dp/dt max obtained by hemodynamic evaluation. LDH, Lactate dehydrogenase; CK-MB, creatine kinase-MB; AAR, area at risk; LV, left ventricle; MPO, myeloperoxidase; IL-1α, interleukin-1α; TNF-α, tumour necrosis factor-α; IP7, inositol pyrophosphate 7; LVEF, Left ventricular ejection fraction; LVESV, Left ventricular end-systolic volume; LVEDV, Left ventricular end-diastolic volume. *P < 0.01 *versus* Sham, ^#^*P* < 0.01 *versus* I/R, ^§^*P* < 0.01 *versus* OSM.

To elucidate the impact of OSM on cardiac I/R injury in the genetically OSM receptor Oβ free situation in diabetic mice, the Oβ^−/−^ and its littermate control mice (Oβ^+/+^) were injected by STZ to construct diabetes model and were subjected to cardiac I/R injury. Less I/R injury was manifested by lower levels of serum LDH and CK-MB in Oβ^+/+^ mice (Fig.2A and B). With similar AAR/LV, the infarct size was significantly smaller in Oβ^+/+^ mice as compared with Oβ^−/−^ mice (Fig.2C–E). The diabetic Oβ^+/+^ mice exhibited reduced MPO activity (Fig.2F), decreased release of IL-1α and TNF-α (Fig.2G and H) and decreased production of IP7 (Fig.2I) after I/R injury. In parallel, we observed improved cardiac function, as indicated by elevated LVEF, decreased LVESV and LVEDV in Oβ^+/+^ mice (Fig.2J–M). This protection was also demonstrated by increased ±LV dp/dt max (Fig.2N and O) in Oβ^+/+^ mice as compared with Oβ^−/−^ mice. These results suggest that OSM plays an important role in reducing myocardial I/R injury by inhibition of IP7 production.

### OSM inhibits cardiomyocyte apoptosis induced by cardiac I/R injury in db/db mice by reducing IP7 production

**Figure 3:**
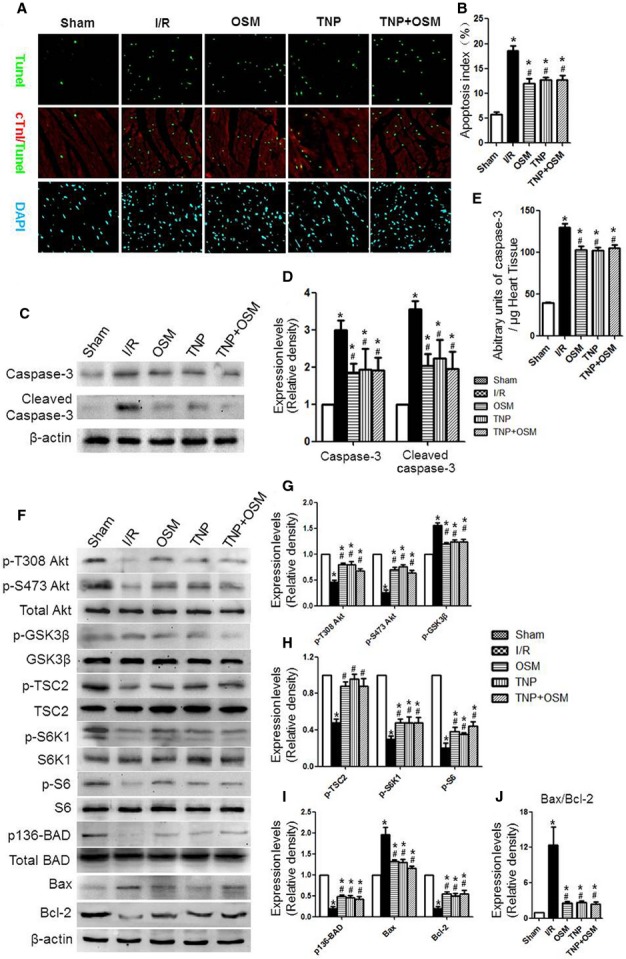
OSM inhibited cardiomyocyte apoptosis induced by cardiac I/R injury in db/db mice by reducing IP7 production. (A and B) Analysis of TUNEL-positive cardiomyocytes. (C and D) Caspase-3 and cleaved caspase-3 expression. (E) Caspase-3 activity. (F–I) Western blot analysis of p-T308 Akt, p-S473 Akt, Akt, p-GSK3β, GSK3β, p-TSC2, TSC2, p-S6K1, S6K1, p-S6, S6, p136-BAD, BAD, BAX and Bcl-2. (J) Calculated Bax/Bcl-2 ratio. *P < 0.01 *versus* Sham, ^#^*P* < 0.01 *versus* I/R, ^§^*P* < 0.01 *versus* OSM.

TUNEL-positive cardiomyocytes were less frequently observed in the OSM group as compared with the I/R group. Similarly, TNP treatment remarkably decreased the number of TUNEL- positive cardiomyocytes compared with the I/R group (Fig.3A and B). Concomitantly, caspase-3 activity determined by a caspase colorimetric assay, caspase-3 and cleaved caspase-3 expression evaluated by Western blot was down-regulated by OSM, TNP or TNP+OSM treatment (Fig.3C–E).

After 3 hrs of reperfusion, western blot analysis revealed that OSM or TNP treatment increased the phosphorylation state of Akt (T308, S473), TSC2, S6K1, S6 and BAD while decreased the phosphorylation state of GSK3β. Furthermore, OSM or TNP administration decreased Bax/Bcl-2 ratio in cardiac tissue that were exposed to I/R injury (Fig.3F–J).

### Oβ knockout enhances cardiomyocyte apoptosis in diabetic mice who underwent cardiac I/R injury

**Figure 4:**
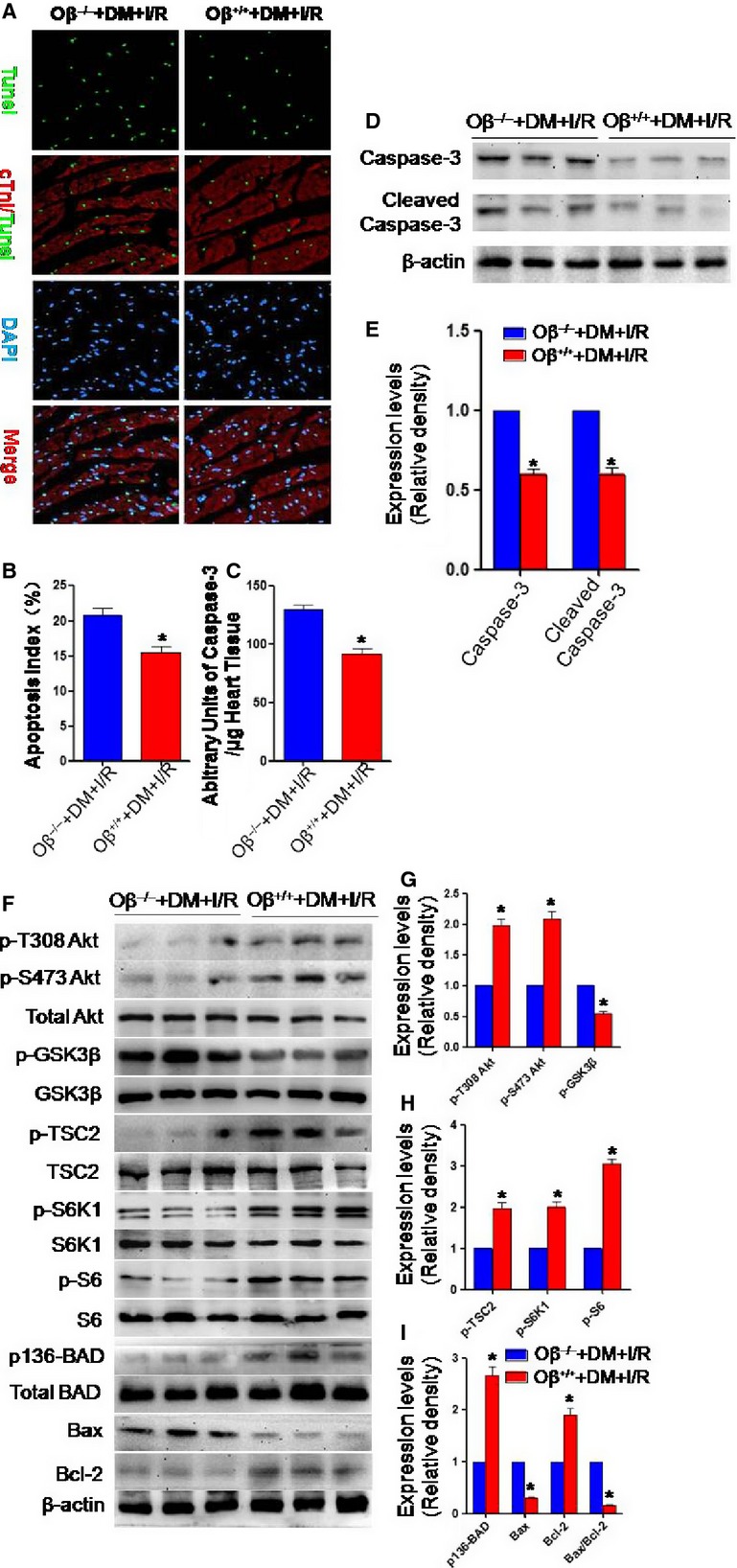
Oβ knockout enhanced cardiomyocyte apoptosis in diabetic mice who underwent cardiac I/R injury. (A and B) Diabetic Oβ^−/−^ mice who underwent cardiac I/R injury had increased TUNEL-positive cardiomyocytes. (C and D) Diabetic Oβ^−/−^ mice exhibited increased caspase-3 and cleaved caspase-3 expression when subjected to I/R injury. (E) Caspase-3 activity was increased in the Oβ^−/−^ group. (F–I) Diabetic Oβ^+/+^ mice exhibited much higher the phosphorylation state of Akt (T308, S473), TSC2, S6K1, S6, BAD. (J)The pro- to anti-apoptotic protein (Bax/Bcl-2) ratio was increased in the Oβ^−/−^ group. *P < 0.01 *versus* Sham, ^#^*P* < 0.01 *versus* I/R, ^§^*P* < 0.01 *versus* OSM.

TUNEL-positive cardiomyocytes, caspase-3 and cleaved caspase-3 expression were remarkably reduced in diabetic Oβ^+/+^ mice who underwent cardiac I/R injury as compared with the Oβ^−/−^ group (Fig.4A–E). Diabetic Oβ^+/+^ mice who underwent cardiac I/R injury exhibited much higher the phosphorylation state of Akt(T308, S473), TSC2, S6K1, S6, BAD and decreased the phosphorylation state of GSK3β. The pro- to anti-apoptotic protein (Bax/Bcl-2) ratio was significantly decreased in the Oβ^+/+^ group (Fig.4F–I).

### OSM improves mitochondrial biogenesis and mitochondrial function in db/db mice who underwent cardiac I/R injury by reducing IP7 production

**Figure 5:**
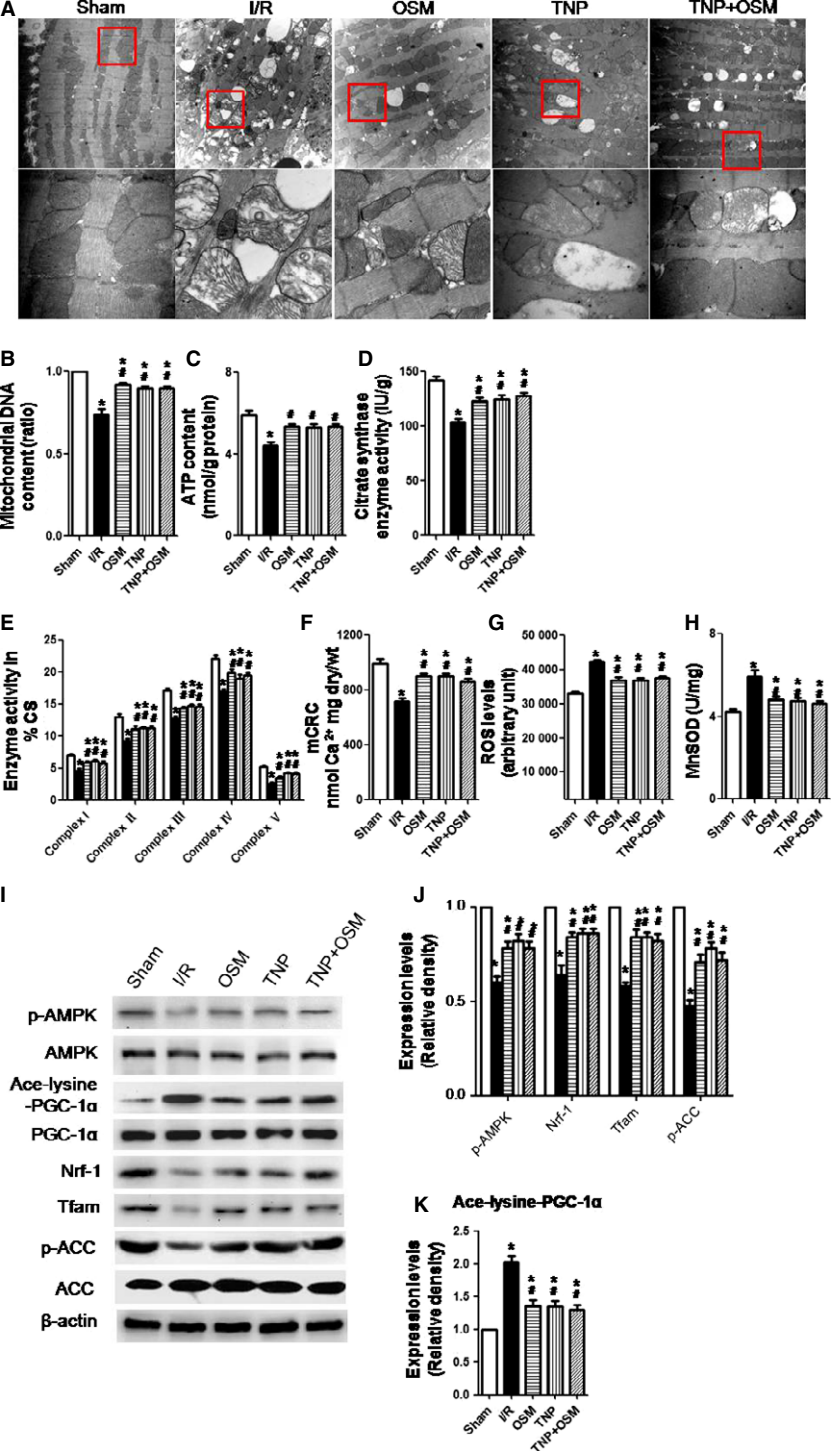
OSM improved mitochondrial biogenesis and mitochondrial function in db/db mice which underwent cardiac I/R injury by reducing IP7 production. (A) Mitochondria morphological defects (magnification: upper panel ×10,000; lower panel ×40,000). (B–E) Mitochondrial DNA content, ATP content, citrate synthase (CS) activity and complexes I/II/III/IV/V activities in the ischaemic myocardium in db/db mice subjected to cardiac I/R injury. (F) Sensitivity of the mitochondrial permeability transition pore (mPTP) opening to calcium as demonstrated by mCRC measurement. (G) ROS levels assessed by EPR spectroscopy. (H) Mitochondrial MnSOD activity. (I–K) Western blot analysis of p-AMPK, AMPK, p-ACC, ACC, Ace-lysine-PGC-1α, PGC-1α, Nrf-1 and Tfam expression. mCRC, mitochondrial calcium retention capacity; MnSOD, manganese superoxide dismutase; AMPK, adenine mononucleotide protein kinase; Nrf-1, nuclear respiratory factor 1; Tfam, mitochondrial transcription factor A; PGC-1α, peroxisome proliferator-activated receptor-γ coactivator-1α. *P < 0.01 *versus* Sham, ^#^*P* < 0.01 *versus* I/R, ^§^*P* < 0.01 *versus* OSM.

Transmission electron microscopy revealed that OSM or TNP treatment alleviated disorganized mitochondria architecture in db/db mice which underwent cardiac I/R injury (Fig.5A). Mitochondrial DNA content (Fig.5B), ATP content (Fig.5C), CS activity (Fig.5D), and complex I/II/III/IV/V activities (Fig.5E) in the ischaemic myocardium were significantly enhanced in the OSM or TNP treated group as compared to the I/R group. OSM or TNP administration increased mCRC (Fig.5F), indicating that sensitivity to calcium-induced mPTP opening was decreased. ROS levels (Fig.5G) assessed by EPR spectroscopy and mitochondrial MnSOD activity (Fig.5H) were decreased in the OSM and TNP treated group as compared to the I/R group.

Western blot analysis revealed that OSM or TNP administration increased the phosphorylation state of adenine mononucleotide protein kinase (AMPK) and acetyl-CoA carboxylase (ACC), the expression of Nrf-1 and Tfam. The acetylation state of PGC-1α was decreased in the OSM or TNP treated group (Fig.5I–K).

### Oβ knockout impairs mitochondrial biogenesis and function in diabetic mice who underwent cardiac I/R injury

Oβ knockout mice exhibited increased mitochondria morphological defects (Fig. S1A). The Oβ^−/−^ group showed decreased Mitochondrial DNA content (Fig. S1B), ATP content (Fig. S1C), CS activity (Fig. S1D) and complex I/II/III/IV/V activities (Fig. S1E) as compared to the Oβ^+/+^ group. The mCRC was decreased (Fig. S1F), ROS levels (Fig. S1G) and mitochondrial MnSOD activity (Fig. S1H) were increased in the Oβ^−/−^ group as compared to the Oβ^+/+^ group. Together, these results suggested that Oβ knockout impaired mitochondrial biogenesis and mitochondrial V dysfunction in diabetic mice which underwent cardiac I/R injury. Western blot analysis showed that Oβ knockout decreased the phosphorylation state of AMPK and ACC, the expression of Nrf-1 and Tfam. The acetylation state of PGC-1α was increased in the Oβ^−/−^ group (Fig. S1I and J).

### Oβ knockout impairs glucose homoeostasis and insulin sensitivity in diabetic mice which underwent cardiac I/R injury

Oβ knockout led to a marked impairment in GTT. Glucose concentrations during the intraperitoneal GTT were significantly higher in Oβ^−/−^ mice as compared to the Oβ^+/+^ mice (Fig. S2A). The insulin sensitivity evaluated by intraperitoneal ITT manifested that Oβ knockout significantly decreased the blood glucose-lowering effect of injected insulin (Fig. S2B). The Oβ^+/+^ mice required a higher rate of exogenous glucose infusion to maintain euglycaemia, indicating enhanced whole body sensitivity to the insulin infusion (Fig. S2C). Consistently, Oβ^−/−^ mice exhibited significantly decreased glucose disposal rate (Fig. S2D). Oβ knockout decreased insulin-stimulated glucose transport activity in the soleus, gastrocnemius and epididymal white adipose tissue (Fig. S2E). To obtain corroborative evidence of insulin sensitivity in muscle, we investigated insulin signalling by analysing the tyrosine phosphorylation state of IRS1, the phosphorylation state of Akt (p-T308, p-S473) and GSK3β (Fig. S2F). Western blot analysis revealed decreased phosphorylation state of IRS1 and Akt, increased phosphorylation state of GSK3β in the muscles of Oβ^−/−^ mice (Fig. S2G), further supporting the capacity of Oβ knockout to down-regulate insulin signalling in skeletal muscle.

## Discussion

This study provides the first direct evidence for a role of OSM in regulating apoptosis, mitochondrial biogenesis and insulin sensitivity in diabetic mice which underwent myocardial I/R injury. OSM receptor Oβ knockout exacerbated cardiac I/R injury, increased IP7 production, enhanced cardiomyocyte apoptosis, impaired mitochondrial biogenesis, glucose homoeostasis and insulin sensitivity in diabetic mice which underwent cardiac I/R injury. Interestingly, inhibition of IP7 production by TNP exhibited similar effects as OSM, the combination of OSM and TNP was comparable to each agent alone, indicating a causative role of OSM/IP7 signalling in cardiac I/R injury in diabetic mice. The mechanism of OSM on cardiac I/R injury in diabetic mice is partly associated with IP7/Akt and AMPK/PGC-1α pathway.

Blocking the cardiomyocyte apoptosis process could prevent the loss of contractile cells, minimize cardiac injury induced by I/R injury and therefore slow down the occurrence of heart failure 23. Our study demonstrated that OSM administration significantly reduced cardiomyocyte apoptosis induced by cardiac I/R injury in db/db mice. The diabetic Oβ^+/+^ mice exhibited decreased cardiomyocyte apoptosis as compared to the diabetic Oβ^−/−^ mice who underwent I/R injury. Furthermore, OSM pre-treatment inhibited IP7 production and TNP administration exhibited similar anti-apoptotic effects as OSM, suggesting that IP7 may serve as an effective downstream molecule of OSM.

It has been reported that PI3K/Akt pathway activation leads to BAD phosphorylation and may thereby suppress cell apoptosis 20. In the present study, the anti-apoptotic effects of OSM and TNP were also associated with activation of PI3K/Akt/BAD pathway. The Bcl-2 family is a key regulator of physiological and pathological apoptosis. The relative ratio of proapoptotic proteins (*i.e*., Bax) to antiapoptotic proteins (*i.e*., Bcl-2) plays a key role in determining cell survival or death. It has been demonstrated that the high ratio of Bax/Bcl-2 is associated with greater vulnerability to apoptotic activation 24. In the present study, OSM or TNP treatment decreased Bax/Bcl-2 ratio. On the contrary, the Diabetic Oβ^−/−^ mice exhibited much higher Bax/Bcl-2 ratio, indicating that OSM may regulate cardiomyocyte apoptosis by IP7/Akt/BAD pathway.

Potential roles of mitochondrial dysfunction in diabetes and cardiac I/R injury have recently emerged from *in vitro* studies. Modulating mitochondrial survival pathways will also affect apoptosis and necrosis 25. In accordance, our study identified significant morphological, biogenesis and function defects in mitochondria after cardiac I/R injury in db/db mice. OSM or TNP administration increased, while OSM receptor Oβ knockout decreased Mitochondrial DNA content, CS activity, complex I/II/III/IV/V activities and ATP production. OSM did not exhibit additional protective effects against mitochondria morphology, biogenesis abnormality and mitochondrial dysfunction when administered after TNP treatment. This indicates that IP7 may serve as a mediator of the effects of OSM.

Mitochondria are the main source of ROS, which are fundamental factors in the development of diabetic complications 9,26,27. Therefore, eradicating mitochondrial ROS has become important to ameliorate complications related with diabetes 25. We found lower levels of ROS production in the OSM or TNP treated group, while Oβ knockout increased ROS production. In addition, the OSM group exhibited a down-regulation of mitochondrial MnSOD activity and a decreased sensitivity of mPTP opening as demonstrated by increased mCRC. Both can be regarded as indirect evidence for decreased mitochondria-targeted ROS. On the contrary, Oβ knockout increased MnSOD activity and sensitivity of mPTP opening.

Adenine mononucleotide protein kinase is a major regulator of mitochondrial biogenesis, which promotes mitochondrial biogenesis through PGC1-α 28. Expression of PGC-1 is reported to be undermined in diabetic and insulin-resistant human subjects, respectively 29. ACC is the downstream signalling substrate of AMPK. Consistent with those interpretations, OSM or TNP administration increased the phosphorylation state of AMPK and ACC, the expression of Nrf-1 and Tfam. The acetylation state of PGC-1α was decreased in the OSM, TNP or TNP+OSM group. The results suggested that OSM may regulate mitochondrial biogenesis by AMPK/PGC-1α pathway.

Insulin resistance is associated with mitochondrial dysfunction 30. Mitochondrial dysfunction, including mitochondrial biogenesis abnormality and excessive oxidative stress, is related to the pathological effects of insulin resistance in different tissues. Modulating mitochondrial function may improve insulin resistance and reduce subsequent cardiac mortality. Insulin signalling activation leads to PI3K activation and several downstream serine kinases, including protein kinase B (Akt), protein kinase C, *etc*., all of which eventually modulate the biological and pleiotropic metabolic actions of insulin 31. In the present study, Oβ knockout led to a marked impairment in glucose tolerance and whole body insulin sensitivity in diabetic mice which underwent cardiac I/R injury. Euglycemic-hyperinsulinemic clamp experiments demonstrated decreased glucose disposal in peripheral tissues in Oβ knockout mice. To investigate the underlying mechanism of Oβ knockout in impairment of insulin sensitivity in muscle, insulin signalling was analysed. Western blot analysis revealed decreased tyrosine phosphorylation state of IRS1, decreased the phosphorylation state of Akt(T308, S473) in the muscles of Oβ^−/−^ mice, further supporting the capacity of Oβ knockout to down-regulate insulin signalling. The effects of OSM and TNP in protecting against cardiac I/R injury in diabetic mice were investigated in db/db mice. For the Oβ^−/−^ and Oβ^+/+^ mice, we injected STZ and fed the mice with high glucose and high fat diet to induce diabetic model. Interestingly, the results were consistent between those two animal models.

## Conclusions

This study provides the first direct evidence for a role of OSM in regulating cardiomyocyte apoptosis, mitochondrial biogenesis and insulin sensitivity in diabetic mice undergoing myocardial I/R injury. The effects of OSM are partly associated with IP7/Akt and AMPK/PGC-1α pathway. Moreover, our results suggest that OSM is a potential novel therapeutic target for preventing myocardial I/R injury in diabetic mice.
