# Supplementary material for: Oncostatin M (OSM) protects against cardiac ischaemia/reperfusion injury in diabetic mice by regulating apoptosis, mitochondrial biogenesis and insulin sensitivity
Source: J Cell Mol Med. 2015 Mar 8;19(6):1296–307. doi: 10.1111/jcmm.12501 (PMC4459845; doi:10.1111/jcmm.12501)
Supplement: Supplementary file 1 [file jcmm0019-1296-sd1.doc]

**SUPPLEMENTAL MATERIAL**

**Supplemental Methods**

**LDH and CK-MB release evaluation**

Myocardial cellular damage was evaluated by measuring lactate dehydrogenase (LDH) and creatine kinase-MB (CK-MB) activities in plasma. LDH and CK-MB released from ischemic tissue were determined from arterial blood drawn from the carotid catheter 3h after reperfusion. LDH and CK-MB activities were measured with commercial reagent kits which had adapted to spectrophotometric Auto analyzer (Sigma, St Louis, MO, USA).

**Determination of tissue myeloperoxidase (MPO), IL-1α and tumor necrosis factor-alpha (TNF-α) activity**

Following the 3 h reperfusion period, tissue sample were taken from the AAR zones for MPO, IL-1α and TNF-α activity analysis as previously described ^1^. The activity of MPO was measured spectrophotometrically at 460nm and expressed as units per 100mg of tissue. The concentrations of IL-1α and TNF-α were measured by enzyme-linked immunosorbent assay (ELISA) according to the manufacturer’s instructions. Values are expressed as pg/mg of total protein.

**Construction of I/R injury animal model and hemodynamic evaluation**

I/R injury animal model was constructed by LAD ligation for 30 min followed by 3h reperfusion as previous described ^1^. In brief, rats were anesthetized with 3% isoflurane. The chest was opened through a left thoracic incision. A 6–0 silk suture slipknot was placed at the distal 1/3 of the left anterior descending artery. The left ventricular pressure (LVP) was measured via a Millar Mikro-tip catheter transducer that was inserted into the left ventricular cavity through the left carotid artery. After 30 min of ischemia, the slipknot was released, and the myocardium was reperfused for 3 h. Sham operated control rats underwent the same surgical procedures except that the suture placed under the left coronary artery was not tied.

**Measurement of Myocardial Infarct Size**

Myocardial Infarct Size was evaluated by Evans Blue/TTC staining as previously described ^1^. Three hours after reperfusion, the ligature around the coronary artery was retied, and 1 ml of 2% Evans Blue dye was injected into the side arm of the aortic cannula. The heart was quickly excised after the dye was uniformly distributed, frozen at -80 ℃ and sliced transversally into 1-mm-thick sections. The slices were incubated in 1% 2, 3, 5-triphenyltetrazolium chloride (TTC, Sigma-Aldrich, St Louis, Mo) for 10 min at 37℃. Evans blue stained areas indicated area-not-at-risk (ANAR). Red parts in the heart, which were stained by TTC, represented for ischemic but viable tissue. Staining negative areas indicated infarcted myocardium. Areas of infarct size (IS) and area-at-risk (AAR) were measured digitally using Image Pro Plus software (Media Cybernetics). IS and AAR were expressed as percentages of the left ventricular area (IS/LV and AAR/LV respectively).

**Determination of Myocardial Apoptosis**

Myocardial apoptosis was determined by terminal deoxynucleotidyl transferase-mediated dUTP-biotin nick end labeling (TUNEL) staining and caspase-3 activity assay as previously described ^1^. TUNEL staining was performed with fluorescein-dUTP (In Situ Cell Death Detection Kit; Roche Diagnostics) for apoptotic cell nuclei and 4’,6-diamidino-2-phenylindole (DAPI) (Sigma) stained all cell nuclei. Additional staining was performed using a monoclonal antibody against Troponin I (cTnI, Santa Cruz) for the identification of myocardium. Apoptotic index (AI) was determined. AI= number of TUNEL-positive myocytes / total number of myocytes stained with DAPI from a total of 40 fields per heart (n=5). All of these assays were performed in a blinded manner. Caspase-3 activity was measured with the ApoAlert Caspase-3 Assay Plate (Clontech, Mountain View, Calif) according to the manufacturer’s instructions. Substrate cleavage was monitored fluorometrically with a SpectraMax Gemini spectrophotometer (Molecular Devices) with excitation and emission wavelengths of 350 and 450 nm.

**Determination of Cardiac Function**

Echocardiography was conducted at 24 h after I/R injury as previously described ^1^. Sedated rats (3% isoflurane) were studied on an echocardiography system (Sequoia Acuson, Siemens; 15-MHz linear transducer). Cardiac dimensions and function were assessed by M-mode echocardiography. Left ventricular end-diastolic diameter (LVEDD) and Left ventricular end-systolic diameter (LVESD) were measured on the parasternal left ventricular long axis view. All measurements represent the mean of 5 consecutive cardiac cycles. Left ventricular end-systolic volume (LVESV), Left ventricular end-diastolic volume (LVEDV) and Left ventricular ejection fraction (LVEF) were calculated by use of computer algorithms. All of these measurements were performed in a blinded manner.

**Radiolabeling and detection of IP7**

Three hours after reperfusion, when the ligature around the coronary artery was retied, 400μCi [^3^H] myoinositol was injected into the border zone of myocardial infarction. One week later, the heart was quickly excised, the left ventricular myocardium was isolated, washed and cut into small pieces. Samples were homogenized, precipitated by TCA (final concentration 8.3%) and centrifuged at 13,000×g for 20 min at 4℃. Inositol phosphates were resolved by HPLC. Levels of IP7 are presented as total counts/min (CPM) to show total IP7 yield.

**Glucose Tolerance Test (GTT) and Insulin Tolerance Test (ITT)**

For intraperitoneal GTT, 2.0 g of D-glucose/kg of body weight was injected after a 16-h fast. For ITT, human recombinant insulin (1.5-5 units/kg of body weight, Invitrogen) was administered intraperitoneally after a 4-h fast. Blood glucose levels were measured immediately before and at indicated time points after injection using a reflectance meter (Accu -Chek, Roche Diagnostics GmbH, Mannheim, Germany).

**Euglycemic Hyperinsulinemic Clamp**

The studies were performed in diabetic Oβ^−/−^ and Oβ^+/+^ mice 24h after I/R injury using the insulin clamp technique (10 milliunits/kg of body weight) in combination with high pressure liquid chromatography purified [3-^3^H]glucose and [^14^C]2-deoxyglucose according to Snyder’s methods (18). In details, overnight fasted mice received a priming dose of HPLC-purified [3-^3^H]glucose (10 μCi) and then a constant infusion (0.1 μCi/min) of label glucose for ~3.5 h. After 1 h of infusion, diabetic Oβ^−/−^ and Oβ^+/+^ mice were primed with regular insulin (bolus 40 milliunits/kg of body weight) followed by a 2-h constant insulin infusion (10 milliunits/kg/min). Using a separate pump, 25% glucose was used to maintain the blood glucose level at 100–140 mg/dl, as determined every 10 min using a glucometer (Roche Diagnostics). Peripheral glucose disposal rates (GDR) and glucose infusion rate (GIR) were then measured from collected plasma. At the end of the clamp, mice were sacrificed, the soleus muscle, gastrocnemius muscle, epididymal white adipose tissue (EWAT) were isolated and were snap frozen in liquid nitrogen. Glucose uptake in different tissues were calculated from plasma 2-[^14^C]deoxyglucose profile fitted with double exponential curve and tissue content of 2-[^14^C]deoxyglucose-6-phosphate.

**Acute Insulin Treatment in Mice**

Twenty-four hours after I/R injury, diabetic Oβ^−/−^ and Oβ^+/+^ mice were fasted for 4 h, anaesthetized, and 25 mU/kg human recombinant insulin (Invitrogen) was administered through the portal vein. Soleus muscle, gastrocnemius muscle and epididymal white adipose tissue (EWAT) were collected 120 s after the injection and immediately stored in liquid nitrogen. Protein was isolated with Trizol reagent (Invitrogen, Carlsbad, Calif) and standard Invitrogen protocols. Protein was then used for Western blotting with primary antibodies against Akt, p-Akt (Thr 308, Ser 473), p-GSK3β (Ser 9), GSK3β, p-ACC (Ser221), ACC (Cell Signaling Technology, Beverly, MA). For detection of tyrosine phosphorylation on IRS1, IRS1 was immunoprecipitated from 1 mg total cell lysate and was blotted with a-p-tyrosine and a-IRS1 antibody.

**Isolation of mitochondria**

Myocardium tissues were taken from the ischemic area 3h after cardiac I/R injury. Tissues were washed three times to remove blood, vessels and adipose tissue. Tissues were then minced thoroughly using scissors in ice-cold isolation buffer (sucrose 250 mmol/L; HEPES 10 mmol/L; EGTA 1 mmol/L, pH 7.4) with 5 mg/ml bovine serum albumin (BSA) and were homogenized with a tissue homogenizer (Ultra-Turrax, IKA, Staufen, Germany). The homogenate was centrifuged at 700 g, 4 °C for 10 min. The supernatant was collected and centrifuged again at 14,000 g, 4 °C for 10 min. The resulting pellet was resuspended in isolation buffer by gentle pipetting and centrifuged at 10,000 g, 4 °C for 5 min. The final pellet was resuspended in isolation buffer. The mitochondria protein concentration was determined with a DC protein assay (Biorad, Hecules, CA, USA) with BSA as standard using the Lowry method.

**Mitochondrial calcium retention capacity (mCRC)**

The mitochondrial calcium retention capacity (mCRC) was determined as the capacity of mitochondria to uptake calcium before permeability transition, to test the sensitivity of the mitochondrial permeability transition pore (mPTP) opening to calcium. The calcium uptake of 100 μg mitochondrial proteins was determined in 1 ml incubation buffer (without EGTA) at 37 °C using glutamate and malate as substrates, in the presence of ADP (400 μmol/L). Calcium green-^5^N (0.5 μmol/L, Invitrogen, Carlsbad, CA, USA) was used as indicator to detect extramitochondrial calcium with a spectrophotometer (Cary Eclipse, Varian, Mulgrave, Victoria, Australia) at excitation and emission wavelengths of 500 and 530 nm, respectively. Pulses of 5 nmol CaCl_2_ were added every minute until calcium was no longer taken up and a rapid increase in calcium green fluorescence was detected. mCRC was expressed as total mitochondrial calcium retention in nmol Ca^2+^/mg protein.

**Transmission electron microscopy (TEM)**

Mice were anesthetizing with 3% sodium pentobarbital (100 mg/kg, IP) 24h after reperfusion. Hearts were rapidly removed and washed with PBS solution. Myocardium tissues were taken from the ischemic area and were cut into a 1 mm tissue mass. Images were taken after fixation, soaking, stepwise alcohol dehydration, displacement, embedding, polymerization, sectioning, and staining and then observed with an electron microscope (JEM-2000EX TEM, Japan) as previously described ^3^. Random sections were taken and analyzed by two technicians blinded to the treatments.

**Western blot evaluation**

Three hours after reperfusion, protein was isolated from homogenized heart tissue with standard Invitrogen protocols (Invitrogen, Carlsbad, Calif). After protein concentration quantitation with the modified Bradford assay (Bio-Rad Laboratories, Hercules, Calif), protein was then used for Western blotting with antibodies against Caspase-3, Cleaved caspase-3, Akt, p-Akt (Thr 308, Ser 473), p-GSK3β (Ser 9), GSK3β, p-TSC2 (Thr 1462), TSC2, p-S6K1 (Thr 389), S6K1, p-S6 (Ser 235/Ser 236), S6, p-BAD (Ser 136), BAD, Bax, Bcl-2, p-AMPK (Thr172), Adenine mononucleotide protein kinase (AMPK), p-ACC (Ser221), ACC, Acetylated-Lysine, Peroxisome proliferator-activated receptor-γ coactivator-1α (PGC-1α), nuclear respiratory factor 1 (Nrf-1), mitochondrial transcription factor A (Tfam) and β-actin (Cell Signaling Technology, Beverly, MA). The blots were visualized with a chemiluminescene system (Amersham Bioscience, Buchinghamshire, UK). The signals were quantified by Image Pro Plus software (Media Cybernetics).

**References**

**1. Sun D, Huang J, Zhang Z, *et al*.** Luteolin limits infarct size and improves cardiac function after myocardium ischemia/reperfusion injury in diabetic rats. *PloS one.*2012;7:e33491.

**2. Chakraborty A, Koldobskiy MA, Bello NT, *et al*.** Inositol pyrophosphates inhibit Akt signaling, thereby regulating insulin sensitivity and weight gain. *Cell.* 2010;143:897-910.

**3. Sun D, Shen M, Li J, *et al*.** Cardioprotective effects of tanshinone IIA pretreatment via kinin B2 receptor-Akt-GSK-3beta dependent pathway in experimental diabetic cardiomyopathy. *Cardiovascular diabetology.*2011;10:4.


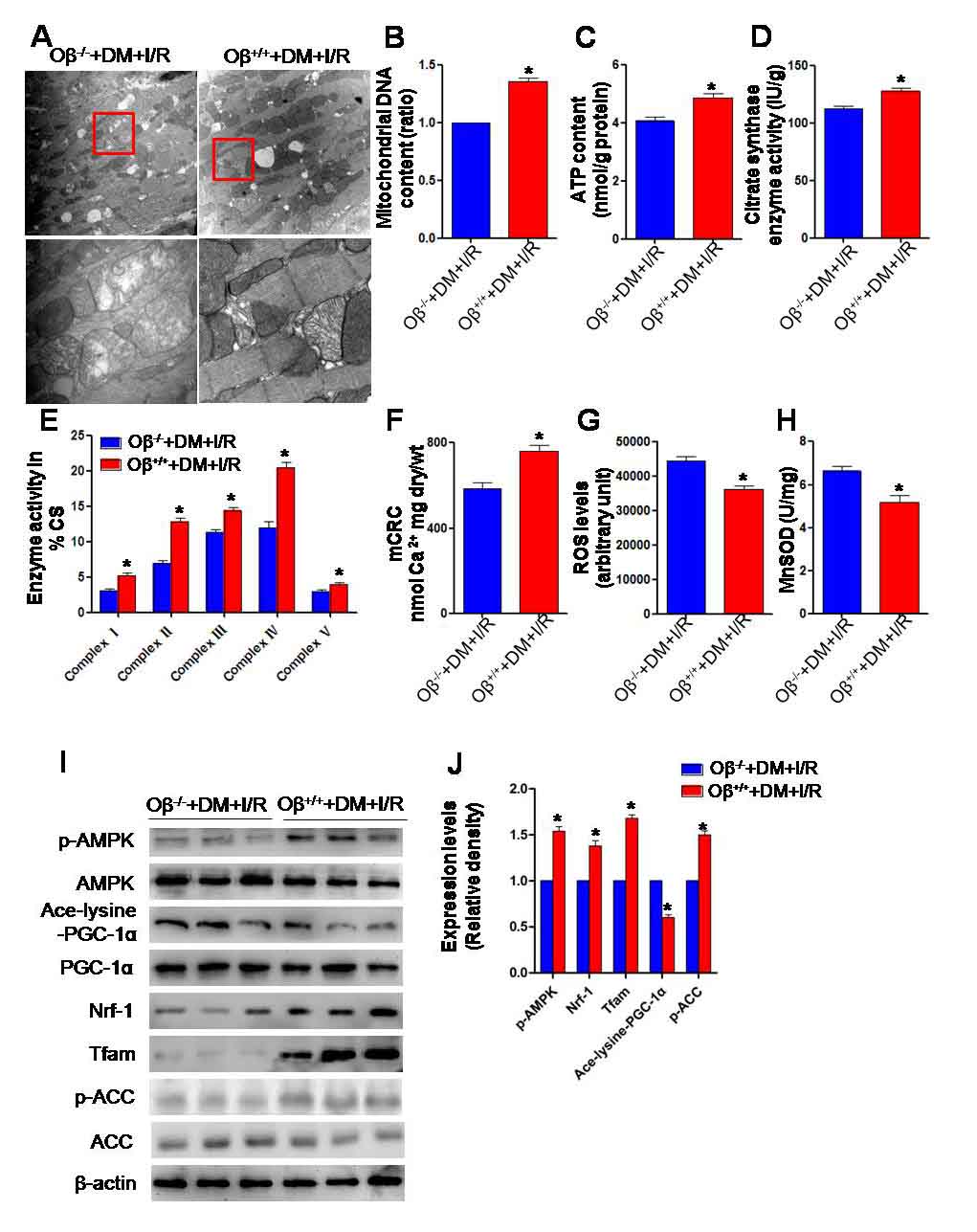


**Figure S1 Oβ knockout impaired mitochondrial biogenesis and function in diabetic mice who underwent cardiac I/R injury**

(A) Oβ knockout mice exhibited severe mitochondria morphological defects (magnification: upper panel ×10000; lower panel ×40000 ). The Oβ^−/−^ group had decreased Mitochondrial DNA content (B), ATP content (C), citrate synthase (CS) activity (D) and complexesⅠ/Ⅱ/Ⅲ/Ⅳ/V activities (E) as compared to the Oβ^+/+^ group. The mCRC was decreased (F), ROS levels (G) and mitochondrial MnSOD activity (H) were increased in the Oβ^−/−^ group. Western blot analysis demonstrated decreased the phosphorylation state of AMPK and ACC, the expression of Nrf-1 and Tfam, and increased the acetylation state of PGC-1α in the Oβ^−/−^ group (I, J). mCRC, mitochondrial calcium retention capacity; MnSOD, manganese superoxide dismutase; AMPK, adenine mononucleotide protein kinase; Nrf-1, nuclear respiratory factor 1; Tfam, mitochondrial transcription factor A; PGC-1α, peroxisome proliferator-activated receptor-γ coactivator-1α. ******p*＜0.01 *vs* Sham, ^#^ *p*＜0.01 *vs* I/R,^§^*p*＜0.01 *vs* OSM.

**
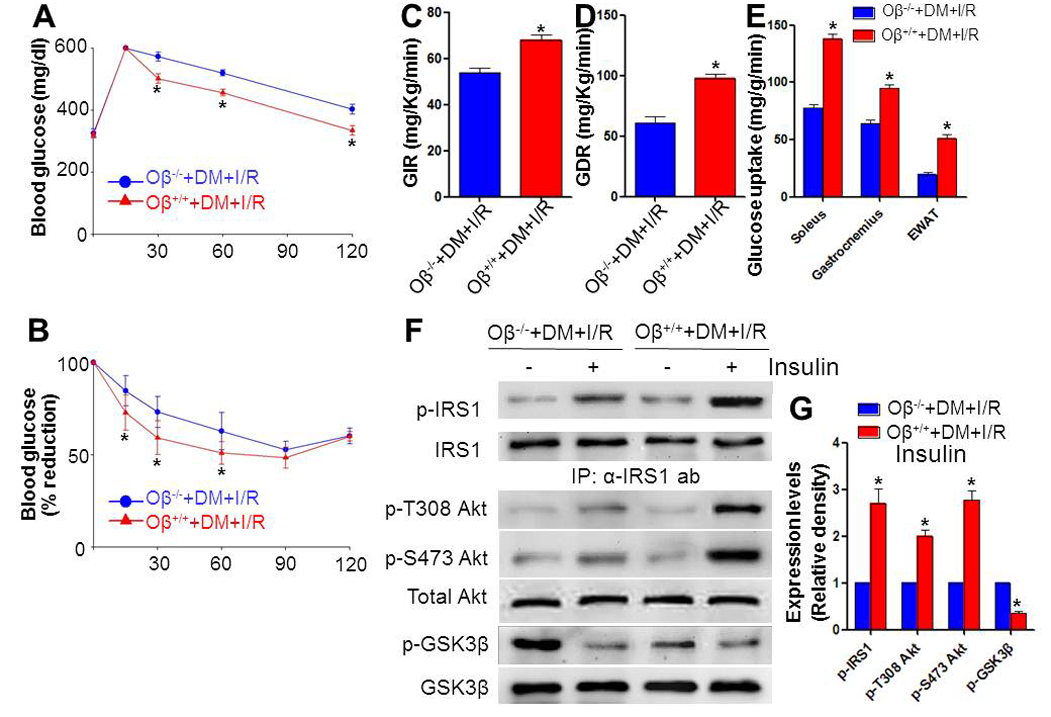
**

**Figure S2 Oβ knockout impaired glucose homeostasis and insulin sensitivity in diabetic mice who underwent cardiac I/R injury**

Oβ knockout led to a marked impairment in GTT (A) and ITT (B). (C) Glucose infusion rate (GIR) was lower in the diabetic Oβ^−/−^ mice subjected to cardiac I/R injury. (D) Oβ^−/−^ mice exhibited significantly decreased glucose disposal rate (GDR). (E) Oβ knockout decreased insulin-stimulated glucose transport activity in the soleus, gastrocnemius and epididymal white adipose tissue (EWAT) as evidenced by euglycemic hyperinsulinemic clamp. (F, G) Insulin signaling analysis showed that Oβ^−/−^ group exhibited decreased tyrosine phosphorylation state of IRS1, decreased the phosphorylation state of Akt (p-T308, p-S473) in diabetic mice underwent cardiac I/R injury. GTT, glucose tolerance test; ITT, insulin tolerance test. **p*＜0.01 *vs* Sham, ^#^ *p*＜0.01 *vs* I/R,^§^*p*＜0.01 *vs* OSM.
